# Urinary Incontinence Among Community-Dwelling Older Women: An Exploratory Cross-Sectional Characterization of Clinical Features, Urinary Habits, and Perceptions

**DOI:** 10.3390/jcm15208041

**Published:** 2026-10-16

**Authors:** Carla Cárcamo-Cid, Karen García-Tobar, Adolfo Fuentes-Ramirez, Emilia Escalona

**Affiliations:** Instituto de Ciencias Biomédicas, Facultad de Ciencias de la Salud, Universidad Autónoma de Chile, Talca 3467987, Chile

**Keywords:** urinary incontinence, pelvic floor muscle, healthy aging, ICIQ-SF

## Abstract

**Background/Objective:** Urinary incontinence (UI) negatively affects wellbeing. As it is often seen as a normal part of aging, management is frequently delayed or ignored, challenging healthy aging. This study aimed to characterize UI in community-dwelling older women by assessing sociodemographic aspects, symptoms, pelvic floor muscle strength, urinary habits, and perceptions regarding UI. **Methods**: We conducted a cross-sectional study in older women from four communities in Chile. After educational sessions on UI, women voluntarily enrolled. We applied the ICIQ-SF, and pelvic floor muscle strength was evaluated using the Modified Oxford Scale. We also collected information on UI-related topics. **Results**: Among 32 older women assessed, 40.6% had stress UI, 3.1% had urgency UI, and 56.3% exhibited mixed UI. The mean ICIQ-SF score was 13.75 ± 3.5, corresponding to severe UI, with a mean score of interference on daily life of 6.75 ± 1.92. Older women exhibited low pelvic floor muscle strength (Oxford: 1.97 ± 1.03), a high prevalence of cystocele (84.4%; *n* = 27), and coexisting fecal incontinence (40.6%; *n* = 13). An association between UI severity and pelvic floor muscle strength was evaluated (Spearman’s ρ = −0.242, *n* = 32), but the weak inverse correlation was not statistically significant (*p* = 0.180). **Conclusions**: Community-dwelling older women with UI exhibited substantial symptom severity accompanied by low pelvic floor muscle strength, pelvic organ prolapse, coexisting fecal incontinence, and potentially modifiable UI-related habits and perceptions. Although these findings derive from an exploratory sample, they highlight opportunities for community-based education, early detection, and pelvic floor health interventions to support healthy aging.

## 1. Introduction

Urinary incontinence (UI) refers to the involuntary leakage of urine. The most common subtype of UI is stress urinary incontinence (SUI), which involves leakage during activities that increase intra-abdominal pressure that impacts pelvic floor pressure, such as coughing, laughing, sneezing, or exercising [1,2]. Urgency urinary incontinence (UUI) is characterized by involuntary urine leakage associated with a sudden, compelling desire to void that is difficult to defer and may be associated with underlying physiological alterations [1,2]. When both SUI and UUI symptoms are present, the condition is called mixed urinary incontinence (MUI), characterized by involuntary urine leakage associated with both physical efforts, such as coughing or sneezing, and urinary urgency [1]. UI symptoms are more common in women, especially in the aging population [3], and are frequently associated with dysfunction or weakness of the pelvic floor muscles, which commonly arise during pregnancy or childbirth, or at the time of menopause [4,5]. Habits and lifestyles can also be key factors [6]. In Chile, UI is demographically variable but highly prevalent. A study conducted among community-dwelling older adults in Vilcún reported a prevalence of 48.1% among individuals aged 60 years and older, with UI being more frequent in women [7]. A previous study in a specific population of Chilean women reported a higher prevalence of 62.2% [8]. More recently, a cross-sectional study reported a similar prevalence of 62.6% among women aged 30–81 years in Chillán [9]. UI leads to significant challenges, including difficulties in daily activities, insomnia, restricted fluid intake that may cause secondary health issues, decreased social participation, loss of autonomy and wellbeing, and negative effects on mental health [10,11,12,13]. However, the impact of UI on daily life still requires more understanding, and sociodemographic and clinical data characterizing Chilean women with this condition remain limited [7,14]. Understanding the severity of UI and its associated clinical and social characteristics is essential for encouraging effective prevention, early diagnosis, and intervention strategies. For this reason, this study aimed to characterize demographic, anthropometric, and health features, as well as pelvic floor muscle strength, pelvic organ prolapse, urinary habits, UI symptoms, and social perceptions regarding UI among a group of community-dwelling older women with UI. We also explored the association between UI severity and pelvic floor muscle strength. This work contributes to the characterization and understanding of UI severity, clinical features, urinary habits, and perceptions among community-dwelling older women with UI and supports the development of new health strategies to decrease the severity of UI symptoms.

## 2. Materials and Methods

### 2.1. Participant Recruitment

This was an exploratory cross-sectional characterization of a community sample of older women with UI recruited from four neighborhood associations in Talca, Region del Maule, Chile. Participants were specifically recruited based on self-reported UI symptoms affecting their daily lives. The study was approved by the Ethics Committee of Universidad Autónoma de Chile (No. CEC 58-25). Recruitment was conducted alongside educational workshops on UI held at community centers where older women regularly gather. These talks addressed female anatomy, clinical aspects of UI, associated risk factors, prevention, and currently available therapeutic approaches. Following these activities, women who reported UI symptoms and expressed interest in participating were invited to undergo eligibility screening. To be included, participants had to be at least 60 years old (1), be able to speak and understand Spanish (2), experience UI with symptoms impacting their daily lives as assessed by the ICIQ-SF (3), obtain a score ≤ 9 on the 15-item Yesavage Geriatric Depression Scale (GDS-15) (4) Ref. [15], and be members of a community centers like a neighborhood association or senior club (5). The GDS-15 cutoff was established to reduce the potential influence of moderate-to-severe depressive symptoms on self-reported daily life impact, as higher depressive symptom burden may independently influence daily functioning and cause poorer quality of life in older adults [16,17]. In Chilean older adults, GDS-15 scores of 10–15 are indicative of established depression [18]. No additional exclusion criteria were applied. Participants who did not meet any of the listed inclusion criteria were considered ineligible for the characterization.

Between December 2025 and April 2026, after educational talks, a total of 41 women over 60 years old expressed interest in participating in this study and provided their informed consent. Eligibility criteria were subsequently assessed. One woman did not meet the eligibility criteria based on the GDS-15 criteria and was guided to receive the appropriated health attention. The severity of UI was assessed using the International Consultation on Incontinence Questionnaire Short Form (ICIQ-SF) [19], which was applied individually to each older adult woman at their community center. This 21-point scale, following the classification proposed by Klovning et al. (2009), categorizes urinary incontinence severity as: 0–5 points, slight; 6–12 points, moderate; 13–18 points, severe; and 19–21 points, very severe [19,20]. One participant did not have UI. Then, participants who met the eligibility criteria were then invited to proceed with the study assessments. At this stage, three women were unable to continue due to time constraints related to their role as primary family caregivers, while four withdrew before the assessments without providing a reason. Therefore, 32 older women sequentially completed ICIQ-SF, General Health and Urinary Clinical History evaluation, the Pelvic Floor Assessment, and the Urinary Health Questionnaire in scheduled sessions.

### 2.2. General Health, Urinary Clinical History and Pelvic Floor Assessment

Gynecological, physical, and pelvic floor muscle evaluation consisted of two stages. The first stage involved a structured interview regarding General Health and Urinary Clinical History, addressing general health, demographic and lifestyle characteristics, and urinary clinical history. The interview included determination of factors associated with UI [21,22], such us body mass index (BMI), closed-ended questions of chronic cough, physical activity, smoking status, in addition to urinary related questions, UI symptoms, and the circumstances in which urine leakage occurred. Also, information about gynecological, urinary and obstetric clinical history was collected.

Regarding urinary symptoms, the physiotherapist asked participants about their daytime and nighttime voiding frequency and whether they experienced a sudden and compelling need to urinate that was difficult to defer, which was explained to participants as urinary urgency. When urgency was reported, participants were also asked how long they were able to defer voiding after the onset of the sensation. The circumstances associated with urine leakage were then systematically assessed using predefined categories, including coughing, sneezing, laughing, walking, exercising, lifting weight, sexual intercourse, and while on the way to the toilet. Participants identified all situations in which they experienced urine leakage. Based on the overall pattern of symptoms reported during the structured interview, the specialized physiotherapist classified UI as SUI, UUI, or MUI. For example, UUI was identified when urine leakage was associated with urinary urgency, including episodes occurring during the day or night, in the absence of leakage associated with physical exertion such as coughing or sneezing. The same specialized kinesiologist conducted the urinary assessment and classified the type of UI for all participants to ensure consistency across evaluations.

The second part included a general physical exam and an assessment of the pelvic floor. Strength of voluntary pelvic floor muscle contraction was measured by the specialized professional using the modified Oxford Scale [23]. This is a widely accepted method for pelvic floor muscle strength assessment which ranges from 0 to 5 a scale for muscle strength [23], where 0 represents the absence of detectable muscle contraction; 1 visible or palpable contraction without active movement; 2 active movement without overcoming gravity or resistance; 3 active movement against gravity but not resistance; 4 full-range movement against gravity and moderate resistance; and 5 indicates normal pelvic floor muscle contraction with active movement sufficient to overcome gravity and resistance [24]. To perform the Modified Oxford Scale assessment, participants were positioned in the supine position with the hips flexed and abducted, knees flexed, and feet together. A cushion was placed under the knees to provide support and allow the lower limbs to remain relaxed. Participants were instructed to perform a voluntary pelvic floor muscle contraction, while minimizing concomitant activation of the abdominal, gluteal, and lower-limb muscles. They were asked to perform three to five pelvic floor muscle contractions, depending on the quality of the contractions and their understanding of how to correctly contract the pelvic floor muscles. The best contraction achieved across all attempts was used to determine the Modified Oxford Scale score [24].

Two pelvic organ prolapses were also evaluated, cystocele which is a protrusion of the bladder into your vaginal canal and rectocele which is the prolapse of the rectum bulging forwards into the vagina [25]. To determine cystocele and rectocele severity Baden–Walker classification was used [25]. Finally, women were directly asked whether they had experienced fecal incontinence, as the involuntary loss of stool. If fecal incontinence was reported, they were asked about the frequency.

### 2.3. Habits and Perceptions Regarding Urinary Incontinence

A urinary health questionnaire developed specifically for this study was administered individually to each participant to collect information on urinary habits, previous knowledge, and perceptions that could contribute to contextualizing UI in this age group. This questionnaire was not standardized or psychometrically validated and was used for descriptive purposes only.

The questions included yes/no questions addressing whether participants restricted fluid intake as a strategy to reduce urine leakage, their perception of UI as part of the aging process, whether they sometimes urinated without sitting on or touching the toilet seat, whether they considered UI a taboo subject of society, whether they had prior knowledge of pelvic floor muscle strengthening as a strategy to reduce UI symptoms, and whether they practiced pelvic floor muscle exercises. If the latter response was affirmative, two additional questions explored the type of exercises performed and their frequency.

Because questions were administered after educational talks, participants were specifically instructed to answer questions concerning previous knowledge and perceptions according to what they knew or believed before participating in the educational session.

### 2.4. Data Analysis

All collected data were tabulated and analyzed anonymously. Demographic data and responses to closed-ended questions were analyzed by frequency and percentages, whereas quantitative variables were summarized using means and standard deviations. Normality was initially explored using the Shapiro–Wilk test. Given the distribution of the data and the ordinal/discrete nature of the Oxford and ICIQ-SF scores, associations between these variables were assessed using Spearman’s rank correlation coefficient (ρ).

Correlation analyses were performed using complete pairs. Statistical significance was evaluated using two-sided permutation tests with 200,000 permutations, and 95% confidence intervals for ρ were estimated using 20,000 paired bootstrap resamples. Statistical calculations were independently performed in Python 3 using NumPy and for visualization of correlation graphs we used JavaScript and React v18.3.1. GraphPad Prism 11.0.2 was used for graphical presentation of symptom-frequency data. Grammarly v.1.1.2 was used for superficial text editing and redaction.

## 3. Results

### 3.1. Study Population General Attributes

The general features of the women in this study are listed in Table 1. The women included were mostly aged between 70 and 79 years (65.6%; *n* = 21), followed by those aged 60 to 69 years (21.9%; *n* = 7). Women aged 80 to 89 were 9,4% (*n* = 3); meanwhile, only one participant was more than 90 years (3.1%). BMI was included because overweight and obesity have been associated with an increased risk of UI [26], as well as smoking and coughing [22]. BMI in the group was mainly in the overweight category (40.6%; *n* = 13), followed by normal weight (31.3%; *n* = 10). Grade I and grade II obesity were identified in 18.8% (*n* = 6) and 6.3% (*n* = 2) of participants, respectively, while underweight was observed in only one older woman (3.1%). A total of 37.5% (*n* = 12) of the women reported being occasional smokers, and 25.0% (*n* = 8) reported having a chronic cough. A total of 68.8% (*n* = 22) of participants said they performed some physical activity, while 31.3% (*n* = 10) said no.

### 3.2. Gynecological and Obstetrical Features of Older Women

The obstetric, UI, and pelvic floor-related aspects of the women of the study are listed in Table 2. The most common type of UI was associated with mixed subtype, affecting 56.3% (*n* = 18) of aged women, followed by SUI at 40.6% (*n* = 13) and UUI in one participant (3.1%). Regarding the ability to hold urine after feeling the need to void, 59.4% (*n* = 19) of women reported being able to retain urine for more than 15 min, while 21.9% (*n* = 7) could do so for 5 to 15 min, and the remaining 18.8% (*n* = 6) indicated being unable to retain urine for more than 5 min. Pelvic organ prolapse assessment showed a high frequency of cystocele (84.4%; *n* = 27). Of those, grade 1 cystocele was observed in 53.1% (*n* = 17) and grade 2 in 31.3% (*n* = 10) of women. Rectocele was less common, observed in 25% of the group, distributed across grade 1 (3.1%; *n* = 1), 2 (18.8%; *n* = 6), and 3 (3.1%; *n* = 1). Fecal incontinence is defined by the unintentional loss of solid or liquid stool [27]. Some women, in addition to UI, declared present fecal incontinence (40.6%; *n* = 13), indicating that older women may suffer from both conditions simultaneously. Regarding urinary tract infections in the previous year, most women (65.6%; *n* = 21) reported none. As for obstetric history, 96.9% of older women (*n* = 31) experienced pregnancy and were mothers. The most frequent number of pregnancies was three (43.8%; *n* = 14), followed by two pregnancies (21.9%; *n* = 7). Most childbirths were vaginal. A part of the group had never undergone a cesarean section (46.9%; *n* = 15), and another had never experienced vaginal birth (31.3%, *n* = 10) One, two, and three cesarean sections were reported by 21.9% (*n* = 7), 18.8% (*n* = 6), and 12.5% (*n* = 4) of women, respectively. Regarding vaginal births, the number of vaginal deliveries was more varied, with 14 women reporting two or more vaginal births. Most women did not experience urinary incontinence during pregnancy (80.6%; *n* = 25) or during the postpartum period (77.4%; *n* = 24).

### 3.3. Situational Triggers of Urinary Leakage Episodes

**Figure 1:**
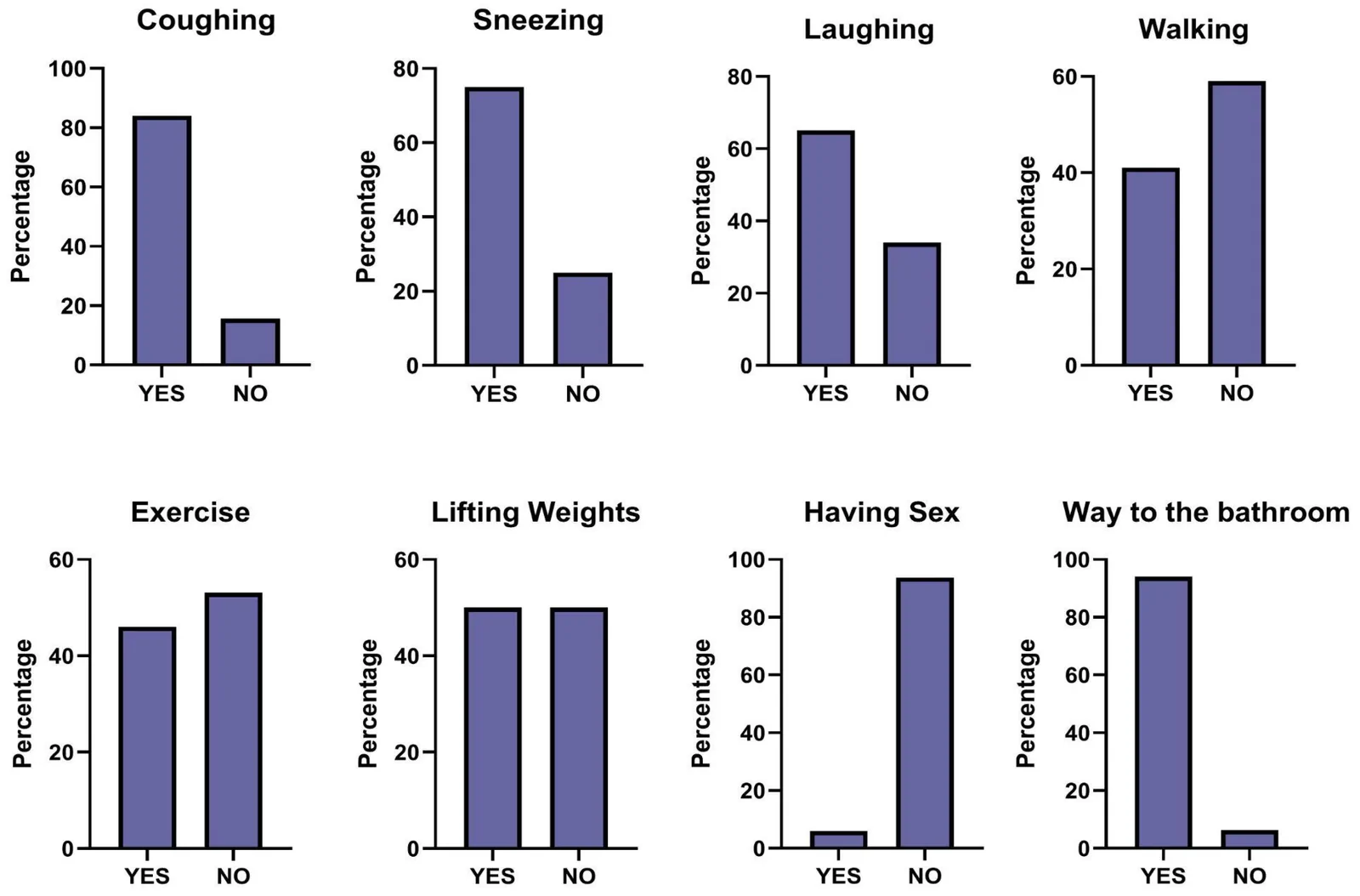
Frequency distribution of urinary leakage episodes according to triggering situation.

Urinary leakage associated with UI may be triggered or occur in a range of situations. Participants identified specific circumstances in which leakage occurred. The most common situation reported for this study group was adult women on their way to the bathroom (93.8%; *n* = 30). It was followed by coughing (84.4%; *n* = 27) and sneezing (75%; *n* = 24) events, which may account for increased episodes of UI during colds or allergies, consistent with the predominant SUI and MUI subtypes present. Laughter was also a common trigger (65.6%; *n* = 21). Half of the participants experienced urine loss when lifting heavy objects (50%; *n* = 16). Less frequent triggers included exercising (46.9%; *n* = 15) and walking (40.6%; *n* = 13). Only a minority reported leakage during sexual intercourse (6.3%; *n* = 2), a finding that may be influenced by the limited engagement in this activity reported by the participants. Overall, these situations were consistent with attributes of the stress and mixed UI predominant in the group. The frequency distribution of these triggers is presented in Figure 1.

### 3.4. Severity of UI and Its Association with Pelvic Floor Muscle Function

Based on the ICIQ-SF in Table 3, most women (40.6%; *n* = 13) reported urine leakage several times a day. Also, most women reported a moderate loss of urine (56.3%; *n* = 18). Regarding the interferences of UI on their daily life, it ranged from 3 to the maximum 10 points, with a mean of 6.75 ± 1.92, indicating an elevated interference on everyday life. According to the ICIQ-SF classification, none of the older women were categorized with mild UI; instead, moderate and severe UI categories were equally common, each affecting 43.8% (*n* = 14) of the women, while 12.5% (*n* = 4) were classified as having very severe UI. The average UI severity score was 13.75 ± 3.5, corresponding to the severe UI category.

Then, strength of voluntary pelvic floor muscle contraction was evaluated using the modified Oxford Scale (Table 3). The average score was 1.97 ± 1.03, showing generally weak pelvic floor muscle contraction [23]. Only two older women (6.3%) achieved a score of 4, identified as having “good” muscle contraction [23], and none obtained the maximum score of 5. Additionally, two participants (6.3%) showed no detectable contraction of the pelvic floor muscles (score 0), indicating significant weakening and deterioration of pelvic floor muscles. These values were consistent with the heightened prevalence of pelvic organ prolapse observed in the women (Table 2).

**Figure 2:**
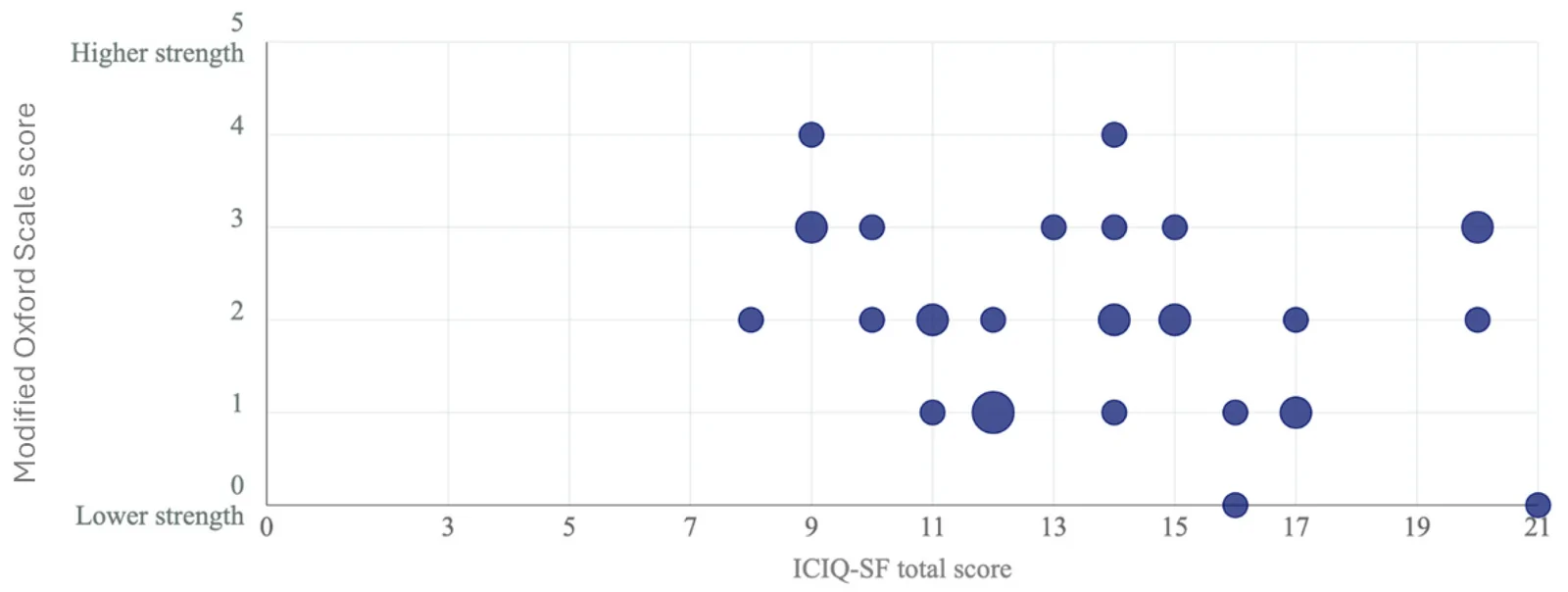
Association between pelvic floor muscle strength and the severity of UI. Individual observations are shown according to the Modified Oxford Scale score and the ICIQ-SF score. Larger points indicate multiple participants with identical scores. Values were analyzed using Spearman’s rank correlation coefficient, resulting in ρ = −0.242, two-sided *p* = 0.180, 95% CI [−0.572, 0.136], *n* = 32.

We also explored the relationship between pelvic floor muscle strength and UI severity using the Oxford and ICIQ-SF scores. As shown in Figure 2, the Spearman correlation coefficient was weak and negative (ρ = −0.242). However, the analysis did not provide statistically significant evidence of an association between pelvic floor muscle strength and UI severity in this group (*p* = 0.180; 95% CI [−0.572, 0.136], *n* = 32).

**Figure 3:**
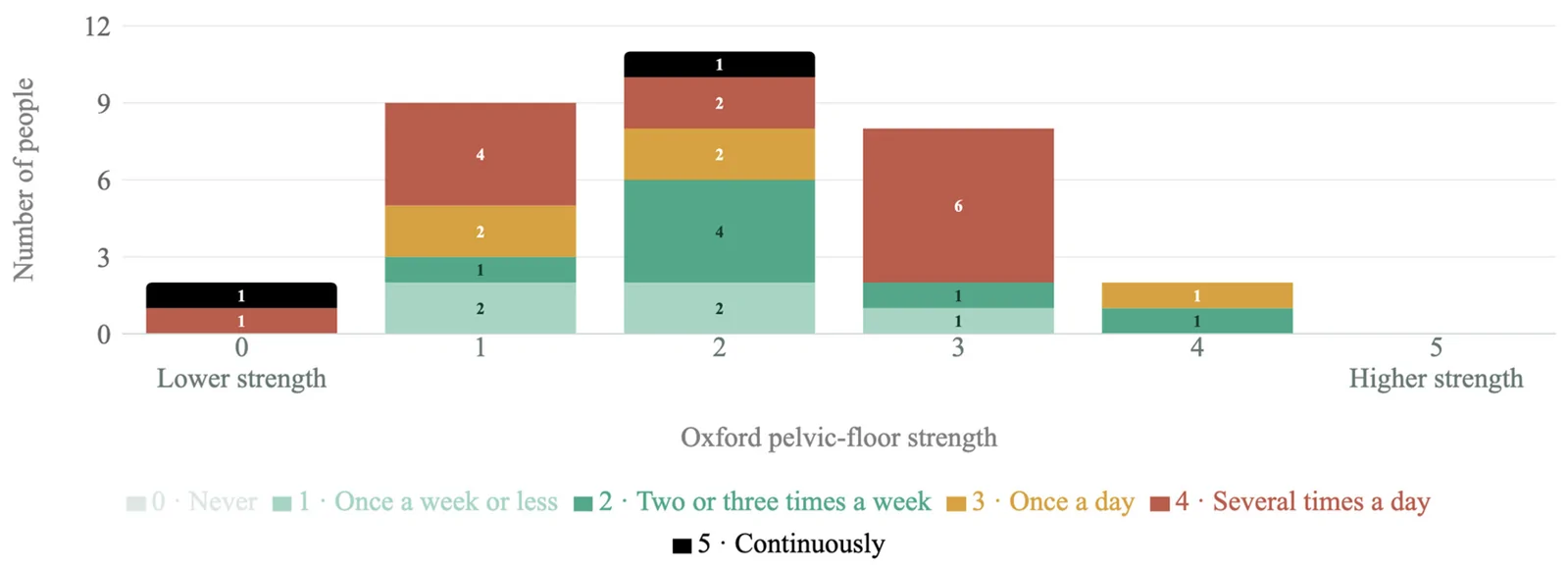
Distribution of urine leakage frequency compared with pelvic floor muscle strength values. Stacked bars represent the number of participants in each ICIQ-SF urinary leakage frequency category according to Modified Oxford Scale score. Spearman’s rank correlation showed no statistically significant association between pelvic floor muscle strength and urinary leakage frequency (ρ = −0.070, two-sided *p* = 0.704; *n* = 32).

Similarly, although the higher frequencies of urine leakage, such as “continuously” were observed in women with lower Modified Oxford Scale score (score 0 and 2) as shown in Figure 3, urine leakage frequencies were distributed across the different Oxford scores, with most observations concentrated around a Modified Oxford Scale score of 2. Spearman’s rank correlation coefficient showed no statistically significant evidence of an association between pelvic floor muscle strength and urine leakage frequency (ρ = −0.070, *p* = 0.704).

**Figure 4:**
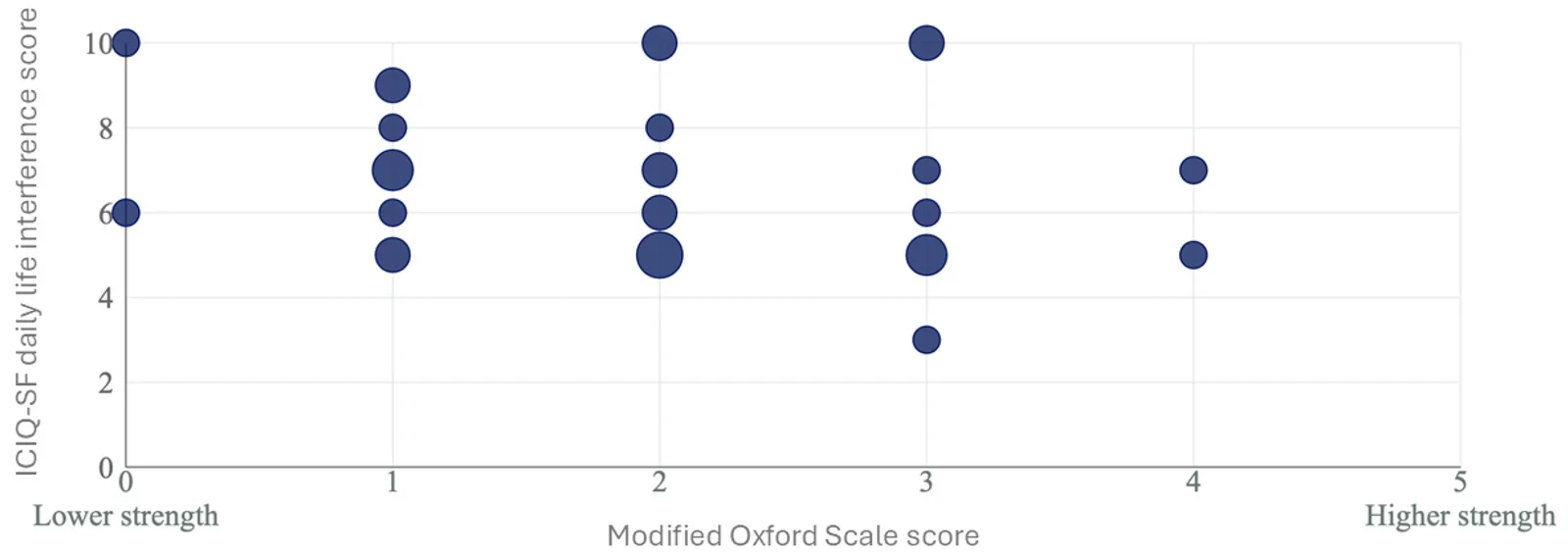
Association between pelvic floor muscle strength and interference of UI with daily life. Individual observations are shown according to the Modified Oxford Scale score and the ICIQ-SF daily life impact score. Larger points indicate multiple participants with identical scores. Spearman’s rank correlation coefficient was ρ = −0.231 (95% CI [−0.551, 0.117], two-sided *p* = 0.201; *n* = 32).

Lastly, we analyzed the association between pelvic floor muscle strength and the degree of interference on their daily life, as assessed by the ICIQ-SF (Figure 4). As the findings presented in Figure 2 and Figure 3, Spearman correlation coefficient between pelvic floor muscle strength and interference with daily life was weak and negative (ρ = −0.231) and not statistically significant (*p* = 0.201; 95% CI [−0.551, 0.117]).

### 3.5. Habits and Perceptions About UI

Certain daily habits among women may be associated with UI symptoms. To gather knowledge regarding a main bad habit associated with UI, particularly common in the female Chilean population, we asked older women whether they sit touching the toilet when urinating. Adopting a non-sitting posture to void can stop the bladder from being fully and properly emptied, which is associated with UI [28,29]. Most women reported non-sitting posture on numerous occasions (81.3%; *n* = 26), especially when using public bathrooms or other bathrooms outside their homes. Another habit investigated was whether women avoided drinking water to prevent urine leakage; 46.9% (*n* = 15) answered yes, exhibiting a higher risk for secondaries conditions. We also evaluated perceptions regarding common myths about UI. A total of 56.3% (*n* = 18) considered UI to be inevitable and a normal aspect of aging. Additionally, 59.4% of participants (*n* = 19) expressed that the topic is taboo in Chilean society. Despite 78.1% (*n* = 25) of women having heard of pelvic floor exercises and their benefits, only 37.5% (*n* = 12) had occasionally practiced them, particularly Kegel exercises or those involving a ball between the thighs. All aging women agreed that education on the subject could have made a major contribution to the prevention and management of their UI. These findings are summarized in Table 4.

## 4. Discussion

This study described UI features and pelvic floor muscle strength in 32 Chilean older women from four community centers. Our cohort was characterized by moderate and severe UI, with more prevalent frequency of urine leakage several times a day, moderate amount of urine leakage, and low pelvic floor muscle strength, with a proportion of women also reporting fecal incontinence (40.6%). We also found a high percentage of women presenting cystocele (84.4%), indicating the coexistence of multiple pelvic floor-related conditions in these women. Cystocele is an anterior vaginal prolapse [30]. This condition is associated with the weakening of the muscles that surround and support the bladder, leading to its descent and further expansion in the anterior vaginal wall. The high prevalence of cystocele observed coexisted with low pelvic floor muscle strength in the group. Although pelvic floor muscle weakness has been associated with pelvic organ prolapse [30], the cross-sectional design and absence of a control group impede conclusions regarding the relationship between these findings in our study. The same consideration should be applied to the frequent use of a non-sitting posture during voiding observed in this community. Although this voiding posture has been associated with UI [28,29], especially for UUI [31], it has also been reported as a prevalent habit among women in the general population [32].

Regarding UI subtypes, consistent with the current findings, Saboia et al. (2019) and Nygaard et al. (2018) also reported mixed and stress UI as among the most common subtypes in the Brazilian populations studied [5,33]. In a Chilean cohort, Neira et al. (2025) reported that 69.4% of older adults with UI had SUI, with women being more frequently affected than men [7]. Similarly, Rincón et al. (2015) reported SUI in 61.3% of Chilean women with UI [9]. In contrast, MUI was the most frequent subtype in our exploratory sample, followed by SUI. This difference should be interpreted cautiously given the limited sample size, recruitment from a single geographical region, and the convenience sampling strategy. Further studies considering differences in demographic, clinical, or lifestyle characteristics across populations could contribute to a better understanding of the variability in UI subtype distribution.

Although UI should not be considered an inevitable consequence of aging, age-related physiological changes in the urinary tract, such as reduced bladder capacity and lower maximum urethral closure pressure, both of which may increase intra-abdominal pressure, may contribute to its occurrence in older women [7]. Female susceptibility is also associated with anatomical characteristics and typical changes related to bladder or pelvic floor muscle dysfunction after menopause, or due to the intrinsic female anatomy [1]. Moreover, in a clinical context, activities that increase intra-abdominal pressure, such as coughing, sneezing, and laughing, may potentiate urine leakage in women with SUI or MUI [34], which were the predominant UI subtypes in our sample.

The fact that 93.8% of participants reported urine leakage while on the way to the toilet could be interpreted as indicative of UUI, but leakage while attempting to reach the toilet should not necessarily be considered equivalent to urgency incontinence. In particular, reporting leakage “on the way to the toilet” describes the circumstances in which the episode occurred but does not necessarily indicate that the participant was unable to reach the toilet because of urinary urgency. UUI is characterized by urine leakage associated with a sudden, compelling desire to void that is difficult to defer [1], and leakage may occur immediately after the onset of urgency. Conversely, in older women, reaching the toilet may involve activities such as standing up from a chair, walking, or climbing stairs, which could potentially contribute to leakage associated with physical exertion. Therefore, the high frequency of leakage reported while on the way to the toilet may reflect different underlying circumstances and should not be interpreted as evidence of UUI alone. Unfortunately, the specific circumstances surrounding these episodes were not further addressed during the interview.

Shang et al. (2023) reported in a meta-analysis that weight-loss interventions, such as bariatric surgery, positively affect quality of life in older women with UI [26]. Overweight is also associated with a sedentary lifestyle, and patients with UI are often reluctant to engage in physical activity, which may further exacerbate the condition [35]. Regular physical activity serves both preventive and therapeutic roles by strengthening the abdominal band and pelvic floor, and modulating neurophysiological mediators, such as stress, involved in UI pathogenesis [35,36]. The majority of aging women in this study were classified as overweight or obese according to their BMI. Both conditions can disrupt normal micturition, increasing UI episodes, because excess weight exerts pressure on the bladder and surrounding muscles, reducing muscle strength and leading to urine leakage [1,7]. As elevated BMI is a risk factor specially for stress and mixed UI [5,37,38] a high proportion of women with overweight or obesity might be expected in the cohort of women with UI. However, other reported risk factors for UI, such as smoking [22] and low levels of physical activity [36], were not predominant among the women evaluated. These findings illustrate the limitations of a descriptive study in establishing relationships between individual risk factors and UI.

History of childbirths is another risk factor for UI. The literature indicates that multiparous women have a higher risk of developing UI, potentially due to atrophy and damage to the pelvic floor muscles during childbirth, including vaginal tears or forceps use, without rehabilitation [39,40]. In terms of delivery type, women who had spontaneous vaginal deliveries have nearly twice the risk of developing stress or mixed UI compared to those who have undergone cesarean or instrumental deliveries [41,42,43]. In the evaluated community sample, 31 of the 32 women had a history of childbirth, with vaginal delivery being the predominant mode of delivery.

The analysis of the correlation between pelvic floor muscle strength and the UI severity or the perceived impact on daily life in our study showed a non-statistically significant weak and inverse association; therefore, the present findings do not provide sufficient evidence of an association between pelvic floor muscle strength and UI severity or daily life impact in this sample. This may be partly explained by the limitations of the study. The sampling strategy specifically designed for this cross-sectional analysis limits the generalizability of the findings; therefore, the results should be interpreted as an exploratory characterization of this community sample rather than as representative estimates for the broader population of older women with UI. The small sample size restricts the statistical power to detect relationships between variables and reduces the precision of the estimates. The absence of a control group also prevented comparisons with older women without UI, limiting the ability to determine whether the characteristics observed in this sample differ from those of older women without UI.

Nevertheless, pelvic floor muscle strength does not appear to be the only factor associated with UI severity, which, according to previous studies, may also be influenced by UI type, years since menopause, constipation, hysterectomy, and chronic conditions such as diabetes mellitus, hypertension, rheumatoid arthritis or hypothyroidism [7,40,44], as well as stress, or help-seeking behaviors [2,45,46], highlighting the multifactorial nature of UI. Furthermore, the highest frequencies of urine leakage among the aging women of this group were not exclusively observed at the lowest levels of pelvic floor muscle strength. This observation is clinically relevant because the Modified Oxford Scale measures voluntary pelvic floor muscle contraction strength. However, continence may also be influenced by factors such as muscle tone, motor control, endurance, other pelvic floor or bladder dysfunctions, and urethral morphology, rather than pelvic floor muscle strength alone [44,47]. These factors may help explain why some women with relatively preserved muscle strength (Oxford scores of 3–4) continued to experience daily and recurrent urine leakage.

Finally, although most women mentioned that they still consider UI a taboo subject in society, they unanimously believed that education about UI could help prevent and manage its symptoms. This finding should be interpreted cautiously, as responses may have been influenced by the educational talk provided before recruitment or by the self-selection of participants. Nevertheless, even considering this potential influence, the positive response suggests that educational initiatives regarding UI may be well accepted by women in this community.

An example of an effective treatment that includes education is multicomponent behavioral training [48]. This approach can improve pelvic floor muscle strength and teach patients strategies to control urinary symptoms through voluntary contraction of the pelvic floor muscles surrounding the urethra. Contracting these muscles repeatedly may promote detrusor relaxation, allowing for greater control over urination and reduced urgency UI [48]. UI should be regarded as a major health problem that needs to be addressed by specialized healthcare professionals, since the global population is getting older and the effect of UI on healthcare systems is expected to grow, not just among women but also for men [3,49]. A community-based screening, education about good urinary habits and interventions such as pelvic floor muscle training could be effective, long-term, and cost-effective preventive and curative alternatives, compared to other options such as laser therapy or surgery [50,51,52], for improving outcomes for aged woman.

## 5. Conclusions

This cross-sectional study showed that UI symptoms can substantially affect the daily lives of this specific sample of community-dwelling older women recruited from four community centers. Most of the women in the study presented characteristics previously associated with UI, such as a history of childbearing, elevated BMI, and low pelvic floor muscle strength. The severity of UI in these older women was reflected by the frequency of daily urinary leakage affecting their everyday life. Although weak inverse correlations were observed between pelvic floor muscle strength and UI severity and the perceived impact on daily life, these associations were not statistically significant. In addition, misconceptions and social perceptions regarding UI were common in this community-dwelling older women, highlighting the potential value of educational initiatives addressing urinary health among older women.

## Figures and Tables

**Table 1:** Features of community-dwelling older women group.

Variable		
Age (Years)	*n*	%
60–69	7	21.9
70–79	21	65.6
80–89	3	9.4
>90	1	3.1
Body mass index (BMI)		
Underweight: <18.5	1	3.1
Normal: 18.5–24.9	10	31.3
Overweight: 25.0 to 29.9	13	40.6
Obesity Class I: 30.0 to 34.9	6	18.8
Obesity Class II: 35.0 to 39.9	2	6.3
Physical Activity		
Yes	22	68.8
No	10	31.3
Smoke		
Yes	12	37.5
No	20	62.5
Chronic cough		
Yes	8	25.0
No	24	75.0

**Table 2:** Clinical, obstetric, and pelvic floor-related characteristics of aged women with UI.

Variable		*n*	%
Urinary Incontinence Types	Stress UI	13	40.6
	Urgency UI	1	3.1
	Mixed UI	18	56.3
Urinary continence	>15 min	19	59.4
	Between 5 and 15 min	7	21.9
	<5 min	6	18.8
Cystocele	No	5	15.6
	Grade 1	17	53.1
	Grade 2	10	31.3
Rectocele	No	24	75.0
	Grade 1	1	3.1
	Grade 2	6	18.8
	Grade 3	1	3.1
Fecal Incontinence	Yes	13	40.6
	No	19	59.4
Urinary tract infections (last year)	0	21	65.6
	1	5	15.6
	2	2	6.3
	3	2	6.3
	>4	2	6.3
Number of pregnancies	0	1	3.1
	1	3	9.4
	2	7	21.9
	3	14	43.8
	4	4	12.5
	5	2	6.3
	7	1	3.1
Number of cesarean births	0	15	46.9
	1	7	21.9
	2	6	18.8
	3	4	12.5
Number of vaginal births	0	10	31.3
	1	8	25.0
	2	8	25.0
	3	4	12.5
	4	1	3.1
	7	1	3.1
Urinary incontinence during pregnancy	Yes	6	19.4 *
	No	25	80.6 *
Postpartum urinary incontinence	Yes	7	22.6 *
	No	24	77.4 *

* Considering the 31 women who had experienced pregnancy.

**Table 3:** Severity of UI and strength of voluntary pelvic floor muscle contraction of older women.

Variable		*n*	%	Min	Max	Mean	SD
ICIQ-SF							
Frequency of leak urine	0—Never	0	0	1	5	3.00	1.24
	1—About once a week or less often	5	15.6				
	2—Two or three times a week	7	21.9				
	3—About once a day	5	15.6				
	4—Several times a day	13	40.6				
	5—All the time	2	6.3				
How much urine do you usually leak?	0—None	0	0	2	6	4	1.34
	2—A small amount	7	21.9				
	4—A moderate amount	18	56.3				
	6—A large amount	7	21.9				
How much does leaking urine interfere with your everyday life?	0–2	0	0	3	10	6.75	1.92
	3	1	3.1				
	4	0	0				
	5	10	31.3				
	6	5	15.6				
	7	7	21.9				
	8	2	6.3				
	9	2	6.3				
	10	5	15.6				
Severity	0–5 Low	0	0	8	21	13.75	3.5
	6–12 Moderate	14	43.8				
	13–18 Severe	14	43.8				
	19–21 Very severe	4	12.5				
Modified Oxford Scale							
Muscle strength	0: No contraction felt	2	6.3	0	4	1.97	1.03
	1: Flicker or trace contraction, minimal or no lift	9	28.1				
	2: Weak contraction, moderate squeeze without a distinct lift	11	34.4				
	3: Moderate contraction with a noticeable inward and upward lift	8	25.0				
	4: Good contraction with a clear lift and resistance against the examiner’s finger	2	6.3				
	5: Strong contraction with a very good, firm lift and sustained pressure	0	0				

**Table 4:** Urinary habits, knowledge, and perceptions regarding UI among older women in the study.

Variable		YES	NO
Sometimes urinates without sitting on the toilet seat	*n*	26	6.0
	%	81.3	18.8
Stop drinking fluids due to UI	*n*	15	17
	%	46.9	53.1
Considers UI a normal part of aging	*n*	18	14
	%	56.3	43.8
Considers UI a taboo subject rarely addressed in society	*n*	19	13
	%	59.4	40.6
Has prior knowledge that pelvic floor muscle strengthening may help reduce UI	*n*	25	7
	%	78.1	21.9
Practices pelvic floor muscle strengthening exercises	*n*	12	20
	%	37.5	62.5
Considers education on pelvic floor care is helpful for UI	*n*	32	0
	%	100.0	0.0

## Data Availability

The datasets presented in this article are not readily available because data are part of an ongoing study. Requests to access the datasets should be directed to corresponding author.

## Abbreviations

The following abbreviations are used in this manuscript:

UI	Urinary incontinence
ICIQ-SF	International Consultation on Incontinence Questionnaire Short Form
BMI	Body mass index
SUI	Stress urinary incontinence
UUI	Urgency urinary incontinence
MUI	Mixed urinary incontinence

## References

[1] Aoki Y., Brown H.W., Brubaker L., Cornu J.N., Daly J.O., Cartwright R. (2017). Urinary incontinence in women. Nat. Rev. Dis. Primers.

[2] Mallah F., Montazeri A., Ghanbari Z., Tavoli A., Haghollahi F., Aziminekoo E. (2014). Effect of Urinary Incontinence on Quality of Life among Iranian Women. J. Fam. Reprod. Health.

[3] Chen X., Jiang S., Guo M., Zhang H., Zhou M., Yao Y. (2026). Gender Disparities in the Prevalence, Trends, and Determinants of Later-Life Urinary Incontinence: A Disproportionate Burden on Women. Aging Med..

[4] Pavlovska O., Savelyeva O., Pavlovska K. (2025). Fluid intake schedule as a possible risk-stratification factor for urinary incontinence in women during menopause. Prz. Menopauzalny.

[5] Nygaard C.C., Schreiner L., Morsch T.P., Saadi R.P., Figueiredo M.F., Padoin A.V. (2018). Urinary Incontinence and Quality of Life in Female Patients with Obesity. Rev. Bras. Ginecol. Obstet..

[6] Bientinesi R., Gandi C., Vaccarella L., Sacco E. (2021). Lifestyle in urology: Benign diseases. Urologia.

[7] Neira-Maldonado C., Coronado J.C., Alvarez M.A., Negron R., Ponce-Fuentes F., Cuyul-Vasquez I. (2025). Prevalence and Associated Factors of Urinary Incontinence Among Chilean Community-Dwelling Older Adults: A Cross-Sectional Study. Cureus.

[8] Salazar A., Oyanedel P., Montiglio C., Campero J.M., Caro C., Monje B., Chavez P., Herrera L. (2005). Prevalence and risk factors of urinary incontinence. Rev. Chil. Urol..

[9] Rincón Ardila O. (2015). Prevalence and risk factors for urinary incontinence among women consulting in primary care, Chile. Rev. Médica Chile.

[10] Nazaripanah N.S., Momtaz Y.A., Mokhtari F., Sahaf R. (2018). Urinary incontinence and sleep complaints in community dwelling older adults. Sleep Sci..

[11] Guler Sonmez T., Ugras E., Gul Sahin E., Fidanci I., Aksoy H., Ayhan Baser D. (2024). The prevalence of incontinence and its impact on quality of life. Medicine.

[12] Pizzol D., Demurtas J., Celotto S., Maggi S., Smith L., Angiolelli G., Trott M., Yang L., Veronese N. (2021). Urinary incontinence and quality of life: A systematic review and meta-analysis. Aging Clin. Exp. Res..

[13] Smith A.P. (2016). Female urinary incontinence and wellbeing: Results from a multi-national survey. BMC Urol..

[14] Aranguiz-Ramirez J., Olivares-Tirado P., Baeza-Correa C., Castells X. (2024). Urinary incontinence in health workers as a predisposing factor for presenteeism in Chile: A mixed-methods exploratory study. Medwave.

[15] Ministerio de Salud de Chile (2018). Orientación Técnica de Atención Integral Para Personas Mayores Frágiles en Unidades Geriátricas de Agudos (Uga) 978-956-348-174-7.

[16] Reichardt L.A., Nederveen F.E., van Seben R., Aarden J.J., van der Schaaf M., Engelbert R.H.H., van der Esch M., Twisk J.W.R., Bosch J.A., Buurman B.M. (2020). The longitudinal association between depressive symptoms and functional abilities in older patients. J. Psychosom. Res..

[17] Yang Y.C., Lin M.H., Wang C.S., Lu F.H., Wu J.S., Cheng H.P., Lin S.I. (2019). Geriatric syndromes and quality of life in older adults with diabetes. Geriatr. Gerontol. Int..

[18] Gallardo-Peralta L.P., Rodriguez-Blazquez C., Ayala-Garcia A., Forjaz M.J. (2020). Multi-ethnic validation of 15-item Geriatric Depression Scale in Chile. Psicol. Reflex. Crit..

[19] Busquets C.M., Serra T.R. (2012). Validación del cuestionario International Consultation on Incontinence Questionnaire Short-Form (ICIQ-SF) en una población chilena usuaria del Fondo Nacional de Salud (FONASA). Rev. Médica Chile.

[20] Klovning A., Avery K., Sandvik H., Hunskaar S. (2009). Comparison of two questionnaires for assessing the severity of urinary incontinence: The ICIQ-UI SF versus the incontinence severity index. Neurourol. Urodyn..

[21] Abufaraj M., Xu T., Cao C., Siyam A., Isleem U., Massad A., Soria F., Shariat S.F., Sutcliffe S., Yang L. (2021). Prevalence and trends in urinary incontinence among women in the United States, 2005–2018. Am. J. Obstet. Gynecol..

[22] Fuganti P.E., Gowdy J.M., Santiago N.C. (2011). Obesity and smoking: Are they modulators of cough intravesical peak pressure in stress urinary incontinence?. Int. Braz. J. Urol..

[23] Zhu H., Zhang D., Gao L., Liu H., Di Y., Xie B., Jiao W., Sun X. (2022). Effect of Pelvic Floor Workout on Pelvic Floor Muscle Function Recovery of Postpartum Women: Protocol for a Randomized Controlled Trial. Int. J. Environ. Res. Public Health.

[24] Laycock J., Jerwood D. (2001). Pelvic Floor Muscle Assessment: The PERFECT Scheme. Physiotherapy.

[25] Persu C., Chapple C.R., Cauni V., Gutue S., Geavlete P. (2011). Pelvic Organ Prolapse Quantification System (POP-Q)—A new era in pelvic prolapse staging. J. Med. Life.

[26] Shang X., Fu Y., Jin X., Wang C., Wang P., Guo P., Wang Y., Yan S. (2023). Association of overweight, obesity and risk of urinary incontinence in middle-aged and older women: A meta epidemiology study. Front. Endocrinol..

[27] Bharucha A.E., Knowles C.H., Mack I., Malcolm A., Oblizajek N., Rao S., Scott S.M., Shin A., Enck P. (2022). Faecal incontinence in adults. Nat. Rev. Dis. Primers.

[28] Palmer M.H., Willis-Gray M.G., Zhou F., Newman D.K., Wu J.M. (2018). Self-reported toileting behaviors in employed women: Are they associated with lower urinary tract symptoms?. Neurourol. Urodyn..

[29] Reynolds W.S., Kowalik C., Kaufman M.R., Dmochowski R.R., Fowke J.H. (2020). Women’s Perceptions of Public Restrooms and the Relationships with Toileting Behaviors and Bladder Symptoms: A Cross-Sectional Study. J. Urol..

[30] Song C., Wen W., Pan L., Sun J., Bai Y., Tang J., Zhong C., Han B., Xia S., Zhu Y. (2023). Analysis of the anatomical and biomechanical characteristics of the pelvic floor in cystocele. Acta Obstet. Gynecol. Scand..

[31] Carvalhais A.G.R., Renato N.J., Margarida F. (2024). Are Toileting Behaviors Associated with Urinary Incontinence Symptoms? An Exploratory Study. Gynecol. Women’s Health.

[32] Yang K.N., Chen S.C., Chen S.Y., Chang C.H., Wu H.C., Chou E.C. (2010). Female voiding postures and their effects on micturition. Int. Urogynecol. J..

[33] Saboia D.M., Firmiano M.L.V., Bezerra K.C., Vasconcelos J.A.N., Oria M.O.B., Vasconcelos C.T.M. (2017). Impact of urinary incontinence types on women’s quality of life. Rev. Esc. Enferm. USP.

[34] Opara J., Czerwinska-Opara W.E. (2014). The prevalence of stress urinary incontinence in women studying nursing and related quality of life. Prz. Menopauzalny.

[35] Leiros-Rodriguez R., Romo-Perez V., Garcia-Soidan J.L. (2017). Prevalence of urinary incontinence and its relation with sedentarism in Spain. Actas Urol. Esp..

[36] Kim M.M., Ladi-Seyedian S.S., Ginsberg D.A., Kreydin E.I. (2022). The Association of Physical Activity and Urinary Incontinence in US Women: Results from a Multi-Year National Survey. Urology.

[37] Faundes A., Miranda L., Cuello M. (2020). Comment on the association between the route of delivery and perineal damage and urinary and fecal incontinence. Rev. Chil. Obstet. Ginecol..

[38] García-Astudillo E., Pinto-García M.P., Laguna-Sáez J. (2015). Incontinence: Frequency and associated factors. Fisioterapia.

[39] González-Maldonado L.A., Erosa-Villarreal R.A., Janssen-Aguilar R., Laviada-Molina H.A., Méndez-Domínguez N.I. (2019). Urinary incontinence: Risk factors and frequency in women above 60 years of age in the Southeast of Mexico. Rev. Mex. Urol..

[40] Demir O., Sen V., Irer B., Bozkurt O., Esen A. (2017). Prevalence and Possible Risk Factors for Urinary Incontinence: A Cohort Study in the City of Izmir. Urol. Int..

[41] Connolly T.J., Litman H.J., Tennstedt S.L., Link C.L., McKinlay J.B. (2007). The effect of mode of delivery, parity, and birth weight on risk of urinary incontinence. Int. Urogynecol. J..

[42] Kjeldsen A.C., Taastrom K.A., Gommesen D., Hjorth S., Axelsen S., Nohr E.A. (2024). Reproductive history of parous women and urinary incontinence in midlife: A National Birth Cohort follow-up study. BJOG Int. J. Obstet. Gynaecol..

[43] Tahtinen R.M., Cartwright R., Tsui J.F., Aaltonen R.L., Aoki Y., Cardenas J.L., El Dib R., Joronen K.M., Al Juaid S., Kalantan S. (2016). Long-term Impact of Mode of Delivery on Stress Urinary Incontinence and Urgency Urinary Incontinence: A Systematic Review and Meta-analysis. Eur. Urol..

[44] Li S., Zhang S., Zhao L., Xiong X. (2025). Effect of Pelvic Floor Muscle Training Combined with Electrical Stimulation Therapy on Stress Urinary Incontinence: A Meta-Analysis. Urol. Int..

[45] Kwon B.E., Kim G.Y., Son Y.J., Roh Y.S., You M.A. (2010). Quality of life of women with urinary incontinence: A systematic literature review. Int. Neurourol. J..

[46] Krhut J., Gartner M., Mokris J., Horcicka L., Svabik K., Zachoval R., Martan A., Zvara P. (2018). Effect of severity of urinary incontinence on quality of life in women. Neurourol. Urodyn..

[47] Vieira G.F., Saltiel F., Miranda-Gazzola A.P.G., Kirkwood R.N., Figueiredo E.M. (2020). Pelvic floor muscle function in women with and without urinary incontinence: Are strength and endurance the only relevant functions? a cross-sectional study. Physiotherapy.

[48] Wyman J.F., Burgio K.L., Newman D.K. (2009). Practical aspects of lifestyle modifications and behavioural interventions in the treatment of overactive bladder and urgency urinary incontinence. Int. J. Clin. Pract..

[49] Vallejos G., Guzmán Rojas R., Valdevenito J.P., Fasce G., Castro D., Naser M., Manríquez V. (2019). Incontinencia Urinaria en el Adulto Mayor. Rev. Chil. Obstet. Ginecol..

[50] Salazar Porras D., Aguilar Hernández L.M., González Alfaro F.J. (2023). Pathophysiology and treatment of urinary incontinence in women. Rev. Medica Sinerg..

[51] Chiang H., Valdevenito R., Mercado A. (2018). Urinary incontinence in elderly adults. Rev. Médica Clínica Condes.

[52] Mazur-Bialy A.I., Kolomanska-Bogucka D., Nowakowski C., Tim S. (2020). Urinary Incontinence in Women: Modern Methods of Physiotherapy as a Support for Surgical Treatment or Independent Therapy. J. Clin. Med..

